# Editorial: Recent advances in platelet-concentrate therapy in regenerative medicine

**DOI:** 10.3389/fbioe.2024.1518095

**Published:** 2024-11-26

**Authors:** Tomoyuki Kawase, Calros Fernando Mourão, Masako Fujioka-Kobayashi, Maddalena Mastrogiacomo

**Affiliations:** ^1^ Division of Oral Bioengineering, Graduate School of Medical and Dental Sciences, Niigata University, Niigata, Japan; ^2^ Department of Basic and Clinical Translational Sciences, Tufts University School of Dental Medicine, Boston, MA, United States; ^3^ Department of Oral and Maxillofacial Surgery, Shimane University Faculty of Medicine, Izumo, Japan; ^4^ Department of Internal Medicine (DIMI), University of Genova, Genova, Italy

**Keywords:** platelet-rich plasma, application, quality control, adjuvant, predictability, biomaterials

## Recent advances in platelet-concentrate therapy in regenerative medicine

PRP has emerged as a widely utilized therapeutic option in clinical settings because of its regenerative properties in various medical fields, including orthopedics, dermatology, and dentistry. Numerous clinical studies have demonstrated the efficacy of PRP, providing compelling evidence for its ability to enhance healing rates, reduce pain, and improve functional outcomes.

However, it is also known that PRP is not a panacea for wounds and trauma. This could be explained by the assumption that PRP is an adjuvant. Thus far, PRP therapy is thought to be predominantly influenced by the quality and treatment protocols of PRP. However, adjuvant theory indicates that the patient’s general and local conditions, as well as the combination of medication or surgical treatments, also influence the success of PRP therapy. However, ironically, this is incompatible with the wishful concept that an ideal drug would work regardless of the patient’s physical condition. Thus, promising preoperative examinations that can appropriately evaluate the patient’s condition (i.e., the levels of responsiveness to PRP) and efficient protocols for PRP treatment (i.e., combinations of supportive biomaterials or bioactive factors) are prerequisites for successful PRP therapy.

In light of such necessities, we proposed this Research Topic and set the mission to collect studies on foresight, rather than completeness. This Research Topic began with an article released by one of the guest editors (Ushiki et al.). This article analyzed body composition and platelet bioenergetics in professional soccer players and proposed a unique strategy for quality control of PRP therapy to overcome the current stagnation by focusing on patients’ body composition and platelet energetics.

Mazzucco et al. reported a unique protocol for the preparation of PRP derivatives (Mazzucco et al.). This article demonstrated that protein-enriched filtered platelet-rich plasma (PEF_PRP_) is effective in the treatment of advanced-stage pressure ulcers in ten patients who could not undergo surgical intervention. The findings indicate that applying biological dressings combined with PEF_PRP_ significantly reduces ulcer size and facilitates optimal scar formation.

Liang et al. summarized in their review article the current situation of PRP therapy for enteric fistula, a serious complication after abdominal surgery, and drew the manner of PRP action while the application to enteric fistula (Liang et al.). Finally, these authors emphasized the necessity of high-quality, well-designed, randomized controlled trials and *in vitro* experiments to improve the quality of evidence.

In a review, Ren et al. focused on how autologous platelet concentrates play a role in each phase of tissue healing and how they work together with different types of biomaterials to participate in tissue regeneration (Ren et al.). Although the standalone application of Autologous Platelet Concentrate (APC) has some shortcomings, the authors discussed articles that have extensively demonstrated that the combination with some biomaterials makes it a complex of molecules capable of synergistically driving the healing process over time. Based on the principle that “simplicity is best,” an emerging approach proposed the association of APC with small doses of nanomaterials to overcome the inherent limitations of an autologous product.

Li et al. focused on the PRP preparation protocol and demonstrated that liquid platelet-rich fibrin (PRF), produced via horizontal centrifugation, positively affects chondrocyte regeneration, particularly through upregulation of anabolic markers like Col2a1 (Li et al.). This effectiveness is almost equal to that of hyaluronic acid (HA) in chondrogenesis and anti-inflammation, suggesting that PRF could be a promising alternative or supplement to HA for the treatment of osteoarthritis and other cartilage-related injuries.

Li et al. aimed to clarify the usefulness of combinational treatment using a developed high-concentration collagen/chondroitin sulfate scaffold and PRP in bone-exposed wounds (Li et al.). These authors found that increasing collagen concentration significantly enhances the mechanical properties of the scaffold and endothelial cell proliferation *in vitro*. These authors further confirmed in a porcine model that this composite material outperforms individual treatments, showing greater vascularization and granulation tissue formation, and suggested the potential of this therapeutic strategy in complex wound treatments, particularly in reconstructive surgeries involving bone-exposed injuries.

Waldmann et al. proposed β-TCP ceramics, commonly used as bone substitute material, as a drug delivery system and potentially applicable to endogenous substances such as growth factors present in blood platelets in tissue regeneration. The authors presented a model of growth factor release from microporous β-TCP ceramics previously loaded with PRP or other blood suspensions (buffy coat and platelet-poor plasma), demonstrating that the biomaterial can represent a drug delivery system because it is based on the activation of PRP after Ca^2+^-triggered activation (Waldmann et al.). This represents a good model for growth factor delivery and is in line with what was discussed in the review by Ren, who saw the biomaterial as a good carrier of growth factors in damaged tissues.

In the last decade, various challenges have been made to optimize PRP preparation, standardize treatment protocols, and significantly advance PRP research and therapy. However, this is not sufficient to achieve the goal. Maximization of the therapeutic potential of PRP is also expected. In dentistry, PRP therapy seems currently not as popular as before. In contrast, PRP therapy has been growing in the fields of sports medicine and orthopedic surgery, both in the sense of medical science and business. The biggest advantage of PRP therapy is the avoidance of surgical invasion and the suppression of the patient’s economic burden. In professional sports, where big money moves, the merit of PRP therapy that minimizes the periods of player withdrawal is immeasurable. However, it cannot predict the response of patients to PRP therapy. To meet athletes’ expectations and increase the predictability of PRP therapy, efforts should be made to combine clinical research with basic science and *vice versa* under a strategy with conviction.

